# Integrated analysis of gut microbiota, serum metabolomics, and proteomics reveals novel associations with clinical symptoms in patients with cerebral infarction

**DOI:** 10.1186/s12866-026-05351-7

**Published:** 2026-06-27

**Authors:** Na Li, Jun Ma, Hui Yan, Jiye Liang, Xinwei Wu, Xiaolin Yu, Mei Lu, Xingbang Wang

**Affiliations:** 1 Department of Dermatology, Jinan Central Hospital, Jinan, Shandong China; 2 Department of Neurology, Yucheng People’s Hospital, Dezhou, Shandong China; 3 Department of Geriatric Medicine, Qilu Hospital of Shandong University, Jinan, Shandong China

**Keywords:** Cerebral infarction, Gut microbiota, Metabolomics, Proteomics, Multi-omics integration, Gut–brain axis, Inflammation

## Abstract

**Background:**

Cerebral infarction (CI) is a major cause of adult disability and mortality worldwide. Mounting evidence supports the critical role of the gut–brain axis in cerebrovascular disease progression. This study aimed to characterize the alterations in gut microbiota, serum metabolome, and serum proteome in patients with CI, and to identify multi-omics signatures associated with clinical symptoms.

**Methods:**

A total of 20 CI patients and 20 healthy controls (HC) were enrolled. Fecal microbiota was profiled using 16 S rRNA gene high-throughput sequencing. Serum metabolomics and proteomics were analyzed using ultra-high-performance liquid chromatography–tandem mass spectrometry (UPLC–MS/MS) and data-independent acquisition (DIA) proteomics, respectively. Spearman correlation and multi-omics integration were applied to explore the associations among microbiota, metabolites, proteins, and clinical indicators.

**Results:**

CI patients displayed significant gut microbiota dysbiosis, with a markedly lower gut microbiota health index (GMHI) and higher microbiota disorder index (MDI) compared with HC (*P* < 0.001). The genera *g_norank_o_RF39* and *Oxalobacter* were significantly enriched in CI patients, whereas *Clostridium_sensu_stricto_1* and *Agathobacter* were enriched in HC. Metabolomic analysis identified 445 differential metabolites, mainly involved in glycerophospholipid metabolism, phenylalanine metabolism, and caffeine metabolism. Proteomic analysis revealed 140 differentially expressed proteins linked to inflammatory responses, calcium signaling, and NF-κB signaling. Multi-omics integration showed that signature gut microbiota was strongly correlated (*P* < 0.005) with key serum metabolites and proteins implicated in CI pathogenesis.

**Conclusions:**

This integrated multi-omics study revealed distinct gut microbiota, serum metabolomic, and proteomic alterations in CI patients. The microbiota–metabolite–protein regulatory axes provide novel insights into the gut–brain axis in CI and may serve as potential diagnostic biomarkers or therapeutic targets.

## Introduction

Cerebral infarction is a leading cause of long-term neurological disability and death, accounting for approximately 6.2 million deaths and 143 million disability-adjusted life years (DALYs) globally in 2019 and places a heavy burden on global healthcare systems [1, 2]. Despite advances in acute recanalization therapies, the molecular mechanisms underlying CI onset and progression remain incompletely understood [3, 4]. The gut–brain axis, a bidirectional communication network between the central nervous system and the enteric nervous system, has emerged as a key regulator of cerebrovascular health [5, 6].

Gut microbiota dysbiosis has been reported to contribute to systemic inflammation, oxidative stress, endothelial dysfunction, and metabolic disturbances, which all represent core pathological processes in CI [7–9]. Serum metabolites produced by gut microbiota, such as short-chain fatty acids, bile acids, and aromatic amino acid derivatives, can enter circulation and modulate brain function [10, 11]. Meanwhile, serum proteomic changes reflect the systemic inflammatory and thrombotic states following CI [12–14]. However, studies simultaneously integrating gut microbiota, serum metabolome, and serum proteome in CI patients remain limited.

This study performed a comprehensive multi-omics analysis to characterize the gut microbiota, serum metabolomic, and proteomic profiles of CI patients. We further explored the correlations among these molecular layers and clinical indicators, aiming to identify potential biomarkers and mechanistic links between the gut microbiota and CI-related phenotypes.

## Materials and methods

### Study population

This study was approved by the institutional ethics committee (KYLL-202204-043-1). All participants provided written informed consent.

### Inclusion criteria

Inclusion criteria were as follows: (1) diagnosed with acute cerebral infarction according to the World Health Organization criteria and confirmed by cranial computed tomography or magnetic resonance imaging; (2) aged 35–75 years; and (3) no antibiotic or probiotic use within 3 months prior to enrollment.

### Exclusion criteria

Exclusion criteria included gastrointestinal diseases, liver or renal failure, malignant tumors, infectious diseases, autoimmune disorders, other neurological diseases, pregnancy or lactation, and inability to provide biological samples. Healthy controls were age-, sex-, and body mass index (BMI)-matched volunteers with no history of cerebrovascular or metabolic diseases.

### Sample collection

#### Fecal samples

Fecal samples were collected from participants using sterile collection tubes during their hospital stay and immediately transported to the laboratory within 2 h. Upon arrival, samples were aliquoted into cryotubes and stored at − 80 °C until analysis.

#### Serum samples

Peripheral venous blood was collected within 24 h of symptom onset, after at least 8 h of fasting, prior to administration of hospital meals. Peripheral venous blood (3 mL) was collected using a coagulation tube, allowed to clot, and centrifuged at 1500 rpm for 10 min at 4 °C. Serum was transferred to cryotubes and stored at − 80 °C.

### Gut microbiota analysis

### DNA extraction and PCR amplification

Microbial DNA was extracted using the E.Z.N.A.® Soil DNA Kit (Omega, USA). DNA quality was examined by 1% agarose gel electrophoresis and quantified using a NanoDrop2000 spectrophotometer (Thermo Fisher Scientific, Wilmington, DE, USA). The V3–V4 hypervariable region of the 16 S rRNA gene was amplified using primers 338 F (5′-ACTCCTACGGGAGGCAGCAG-3′) and 806R (5′-GGACTACHVGGGTWTCTAAT-3′). PCR was performed on an ABI GeneAmp® 9700 thermal cycler (Applied Biosystems, Foster City, CA, USA) using TransStart FastPfu DNA Polymerase (TransGen Biotech, Beijing, China). The thermal cycling conditions were as follows: initial denaturation at 95 °C for 3 min; 27 cycles of denaturation at 95 °C for 30 s, annealing at 55 °C for 30 s, and extension at 72 °C for 45 s; and a final extension at 72 °C for 10 min.

#### Sequencing and bioinformatics analysis

Sequencing was performed on an Illumina MiSeq/NovaSeq platform. Raw reads were filtered using fastp with default parameters (reads containing ambiguous bases or with a quality score < 20 over a 5-bp sliding window were discarded, and reads shorter than 50 bp after trimming were removed). Paired-end reads were merged using FLASH (version 1.2.11) with a minimum overlap of 10 bp and a maximum mismatch ratio of 0.2. Amplicon sequence variants (ASVs) were generated using QIIME2 with Deblur. Taxonomy was assigned against the SILVA 138 database. The Gut Microbiome Health Index (GMHI) was calculated at the genus level using high-confidence ASVs mapped by the SILVA 138 database, based on the ratio of health-associated versus disease-associated genera. This genus-level adaptation makes the index robust to species-level taxonomic misclassification inherent to short-read 16 S amplicon sequencing. Alpha diversity (Chao1, Shannon) and beta diversity (Bray–Curtis, weighted UniFrac) were calculated. Linear discriminant analysis Effect Size (LEfSe) was used to identify discriminative taxa (linear discriminant analysis [LDA] > 2, *P* < 0.05). These analyses were performed using the online tool of Majorbio Cloud Platform (https://cloud.majorbio.com/page/tools/). The use of GMHI and Microbiome Dysbiosis Index (MDI) was pre-specified in our statistical analysis plan, based on their established utility in characterizing gut dysbiosis in human disease. The MDI was calculated as follows, following the established protocol validated by Gunathilake M et al. (Cancers, 2020): MDI = log₁₀ [(total abundance of genera significantly increased in the CI group) / (total abundance of genera significantly decreased in the CI group)] The numerator included g_norank_o_RF39 and Oxalobacter (enriched in CI); the denominator included Agathobacter and Akkermansia (depleted in CI). The differential genera were determined by FDR-corrected LEfSe analysis (adjusted *P* < 0.05).

### Serum metabolomics analysis

#### Sample preparation

Serum samples (100 µL) were extracted with 400 µL acetonitrile: methanol (1:1, *v/v*) containing L-2-chlorophenylalanine (1 µg/mL) as an internal standard. an internal standard. After vortexing and ultrasonication, samples were centrifuged, and supernatants were dried and reconstituted. Quality control (QC) samples were prepared by pooling equal volumes of all sera.

#### UPLC–MS/MS analysis

Metabolites were separated on a Waters HSS T3 column and analyzed using a Triple TOF 5600 system (AB SCIEX) in positive and negative ion modes. Data were processed using Progenesis QI. Metabolites were identified against the Human Metabolome Database (HMDB), the Metabolite and Tandem MS Database (Metlin), and an in-house database. Differential metabolites were defined as variable importance in projection (VIP) > 1 and *P* < 0.05.

### Serum proteomics analysis

#### Protein extraction and digestion

Serum proteins were extracted using lysis buffer (8 M urea, 1% SDS) with protease inhibitors. Protein concentration was determined using a bicinchoninic acid (BCA) assay. Proteins were reduced, alkylated, acetone-precipitated, and digested with trypsin (1:50 enzyme: protein) at 37 °C overnight.

#### DIA proteomic analysis

Peptides were analyzed using a Vanquish Neo UPLC system coupled to an Orbitrap Astral mass spectrometer (Thermo) in DIA mode. Raw data were processed using Spectronaut 18. Differentially expressed proteins were defined as *P* < 0.05 and |fold change| > 1.2.

### Statistical analysis

Continuous data are presented as mean ± standard deviation or median (interquartile range). Between-group comparisons were performed using Student’s t-test for normally distributed data and the Mann-Whitney U test for non-normally distributed data. Categorical data were analyzed using the chi-square test. Pathway enrichment analysis for differential metabolites was performed using MetaboAnalyst 6.0 based on Fisher’s exact test. Both raw P values and Benjamini-Hochberg False Discovery Rate (FDR)-adjusted P values (q values) were reported for all enriched pathways. And for multiple comparisons correction, the Benjamini-Hochberg FDR method was applied across all analyses involving multiple testing, including alpha diversity indices, differential metabolite identification (raw *P* < 0.05 and FDR q < 0.05), differential protein identification (raw *P* < 0.05 and FDR q < 0.05), and Spearman correlation analyses. Only features meeting the FDR-adjusted significance threshold (q < 0.05) are reported in the results. *P* < 0.05 was considered statistically significant.

## Results

### Subjects

Between January 2022 and October 2023, a total of 40 participants were strictly enrolled according to the specified inclusion and exclusion criteria, including 20 patients with cerebral infarction and 20 healthy control subjects. The mean age was 58.4 ± 8.3 years for the cerebral infarction group and 57.5 ± 9.3 years for the healthy control group. The male-to-female ratio was 1:1 in both groups, with 10 males and 10 females in each. There were no statistically significant differences in age, gender, or body mass index (BMI) between the two groups (*p* > 0.05), indicating baseline comparability (Table 1). Among the CI patients, the most common stroke etiology was large artery atherosclerosis (40%, *n* = 8), the median baseline National Institutes of Health Stroke Scale (NIHSS) score was 4 (interquartile range [IQR], 2–7), hypertension (70%, *n* = 14) and diabetes (25%, *n* = 5) were the major comorbidities, and markers of kidney function (creatinine, estimated glomerular filtration rate [eGFR]) were comparable between groups (Table 1).

**Table 1:** Characteristics of the study participants

		IC(*N* = 20)	HC(*N* = 20)	*p*-Value
Sex	Male	10	10	*P* > 0.05
	Female	10	10	*P > 0.05*
Age		58.4 ± 8.3	57.5 ± 9.3	*P* > 0.05
Height(cm)		166.50	167.02	*P* > 0.05
Weight(kg)		60.08	61.32	*P* > 0.05
BMI(kg/m^2^)		22.35	21.98	*P* > 0.05
Stroke etiology (TOAST)				
LAA		40.0%	**—**	***—***
SVO		25.7%	**—**	***—***
CE		14.3%	**—**	***—***
SOE		5.7%	**—**	***—***
SUE		14.3%	**—**	***—***
NIHSS		4	**—**	**—**
Hypertension		70.0%	25.7%	*P* < *0.05*
Diabetes mellitus		25.7%	11.4%	*P* < *0.05*
eGFR		88.3 ± 16.5	92.1 ± 14.8	*P* > 0.05

### Cerebral infarction is characterized by significant microbial dysbiosis and enrichment of specific taxonomic markers

**Fig. 1:**
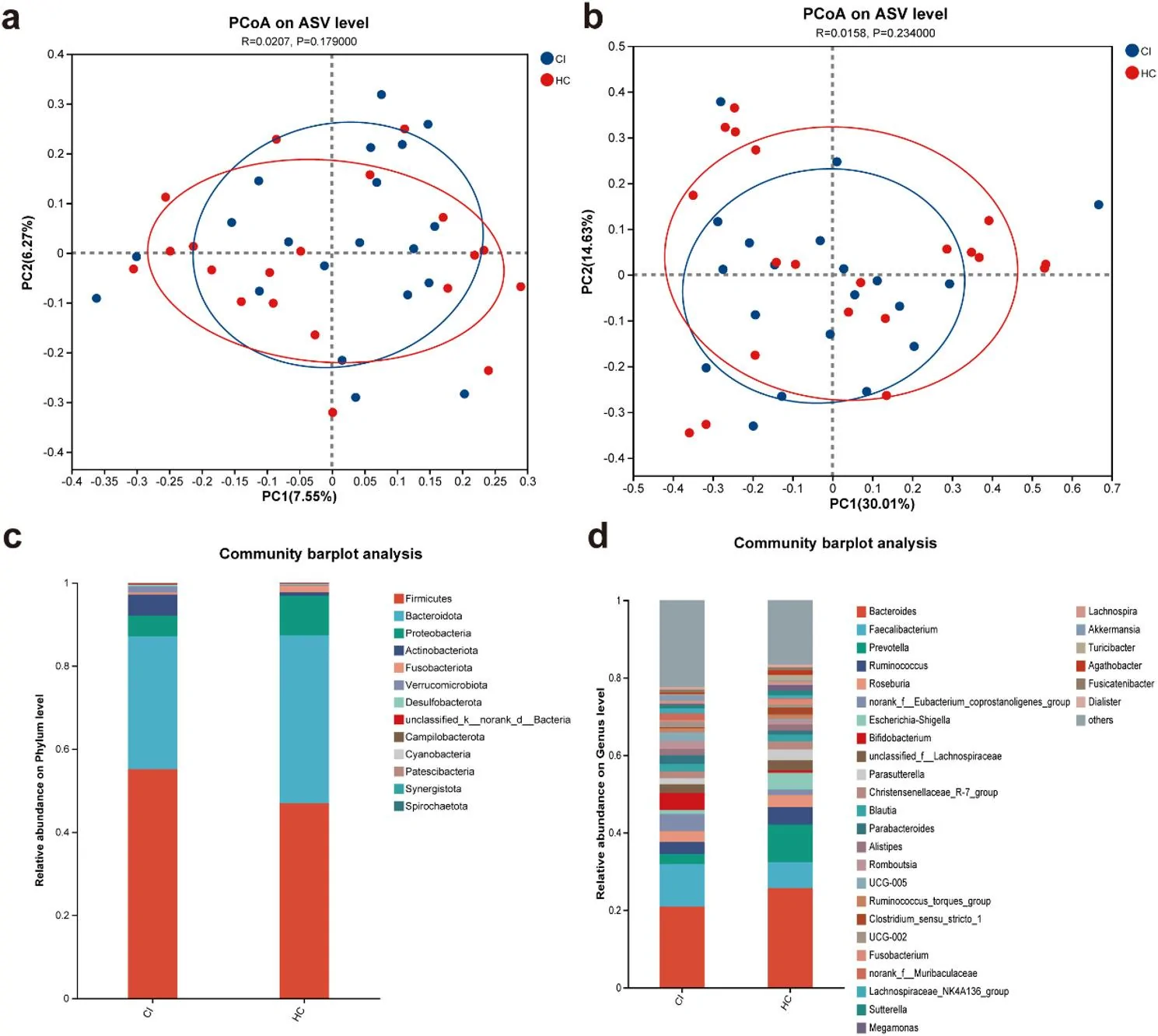
Alterations in the taxonomic profile of gut microbiota at the phylum and genus levels in CI patients. **a** Weighted unifrac PCoA; **b** Unweighted unifrac PCoA. **c**, **d** gut microbiota compositions at the phylum and genus levels

Following high-throughput sequencing of 40 fecal samples, we obtained a total of 2,349,237 high-quality reads. To evaluate the ecological characteristics of the gut microbiota, we first calculated alpha diversity indices. We found that the richness and evenness of the microbial communities, as measured by Ace, Chao, Shannon, and Simpson indices, did not differ significantly between the Cerebral Infarction (CI) patients and Healthy Controls (HC) (*P* > 0.05) (Table 2). Furthermore, we performed beta diversity analysis to assess the overall structural differences between groups; however, Principal Coordinate Analysis (PCoA) plots based on Bray–Curtis and weighted UniFrac distances showed no clear separation, suggesting that the global microbial community structure remained relatively stable in CI patients (Fig. 1a, b).

**Table 2:** The index of α-diversity

	CI	HC	P_value	P_adjust
Ace	476.27 ± 208.00	405.72 ± 135.63	0.3507	0.6256
Chao	464.66 ± 200.34	395.46 ± 130.87	0.3648	0.6256
Sobs	446.8 ± 187.52	384.7 ± 125.17	0.4171	0.6256
Shannon	3.97 ± 0.65	3.86 ± 0.52	0.6168	0.7401
Simpson	0.06 ± 0.05	0.06 ± 0.03	0.7972	0.7972

At the taxonomic level, we observed that the gut landscape was dominated by five major phyla in both groups: Firmicutes, Bacteroidota, Proteobacteria, Actinobacteriota, and Fusobacteriota (Fig. 1c). Consistently, at the genus level, *Bacteroides*, *Faecalibacterium*, *Prevotella*, and *Escherichia-Shigella* were the most predominant taxa (Fig. 1d).

**Fig. 2:**
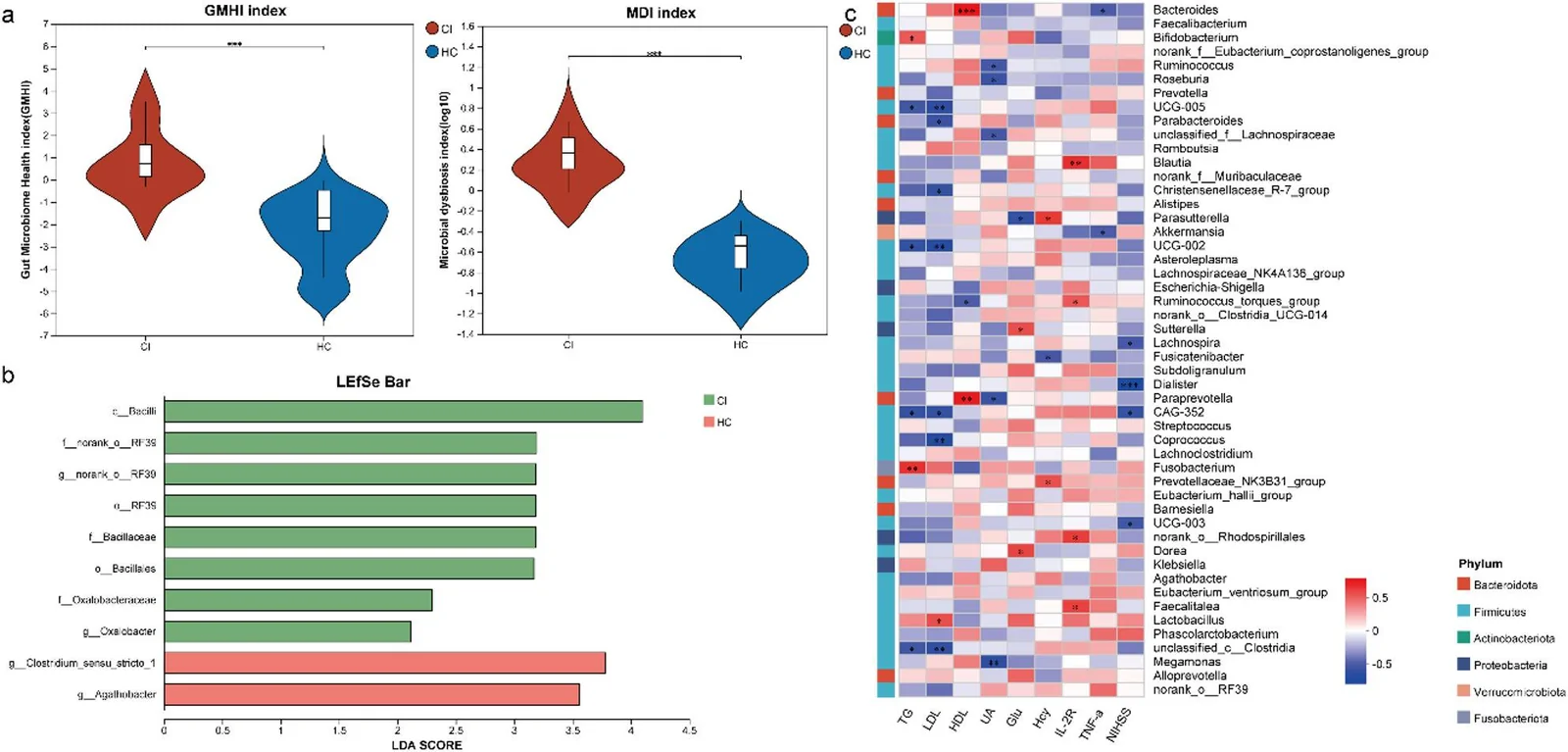
Analysis of gut microbiome health indices, differential taxa, and clinical associations. **a**, Comparison of Gut Microbiome Health Index (GMHI) and Microbiome Dysbiosis Index (MDI) between groups (*P*_adjust< 0.01). **b**, LEfSe analysis identifying significantly enriched genera in CI and HC groups. **c**, Heatmap representing Spearman correlations between differential gut microbiota and clinical indicators (e.g., NIHSS, blood glucose, and lipids). ***P < 0.001; **P < 0.01; *P< 0.05

Despite the similarities in overall diversity, we identified significant shifts in health-related microbial indices. Notably, CI patients exhibited a significantly lower Gut Microbiome Health Index (GMHI) and a higher Microbiome Dysbiosis Index (MDI) compared with the HC group (*P* < 0.001). In addition, FDR correction (Benjamini-Hochberg method) was applied, and both GMHI and MDI remained highly significant, indicating a signature of microbial imbalance in the stroke cohort (Fig. 2a). To pinpoint specific taxonomic contributors to this dysbiosis, we employed Linear Discriminant Analysis Effect Size (LEfSe). Our analysis revealed that *g_norank_o_RF39*, an unclassified genus within the order RF39 (class Mollicutes, phylum Firmicutes), and *Oxalobacter* were significantly enriched in CI patients, whereas *Clostridium_sensu_stricto_1* and *Agathobacter* were characteristically enriched in the HC group (Fig. 2b).

### Specific microbial genera significantly correlate with neurological severity and metabolic dysfunction in CI patients

To explore the potential clinical relevance of these microbial shifts, we performed Spearman correlation analyses between the abundance of specific genera and key clinical indicators. We found that the relative abundances of *Akkermansia*, *Dialister*, and *Lachnospira* were negatively correlated with NIHSS scores, suggesting a potential protective role or an association with milder neurological deficits (Fig. 2c). Furthermore, we identified significant correlations between the gut microbiota and metabolic profiles: *Fusobacterium* was positively correlated with serum triglycerides, *Paraprevotella* showed a positive association with high-density lipoprotein (HDL), and *Sutterella* was positively linked to fasting blood glucose levels (all *P* < 0.05) (Fig. 2c). These findings suggest that gut microbiota alterations in CI patients are closely associated with neurological severity and metabolic profiles, with some microbial taxa correlating with metabolic risk factors (e.g., *Fusobacterium* positively associated with triglycerides) and others with potentially protective lipid profiles (e.g., *Paraprevotella* positively associated with HDL). 

### Serum metabolomic profiling reveals distinct metabolic reprogramming and lipid dysregulation in CI patients

**Fig. 3:**
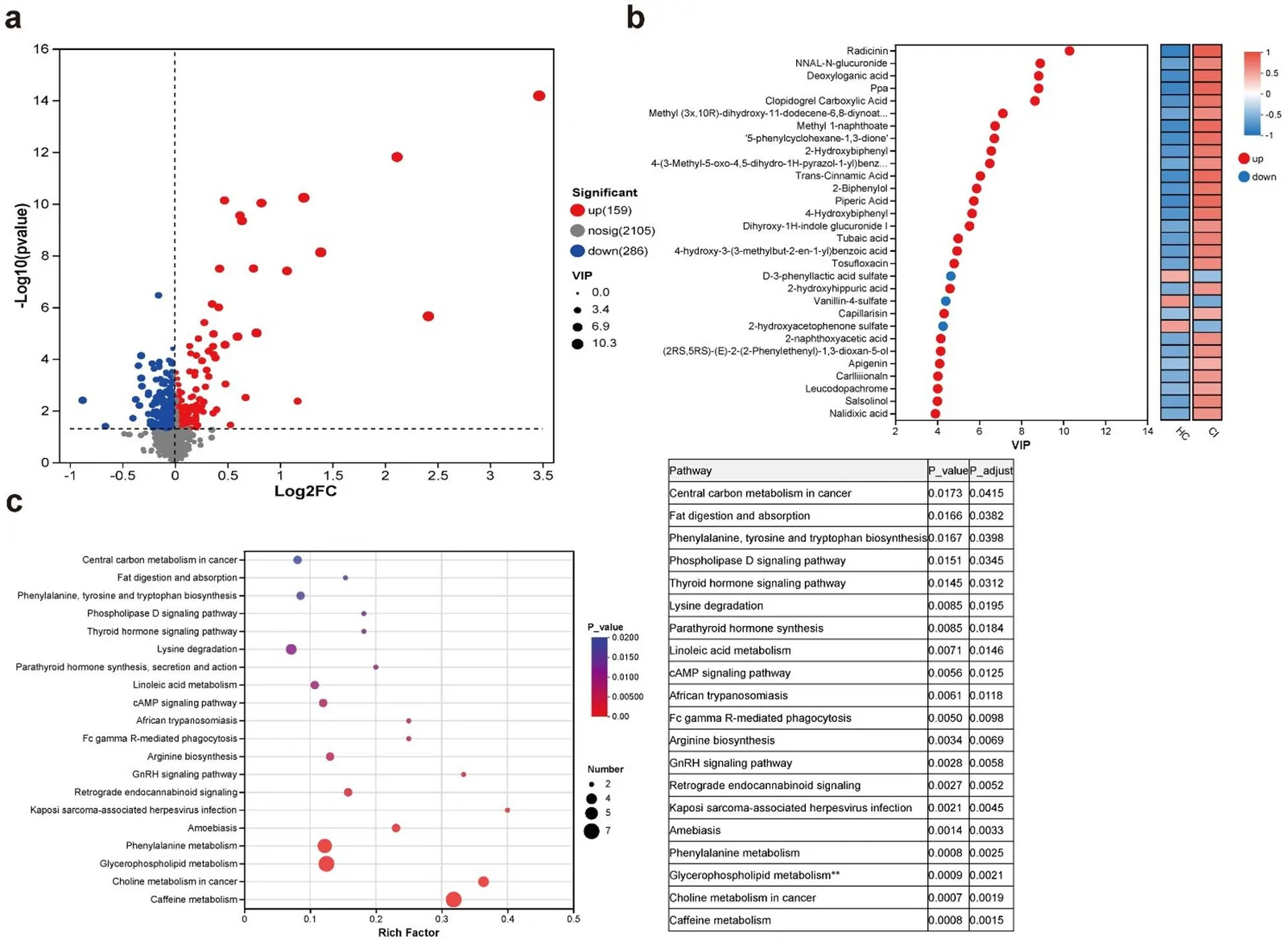
Differential metabolomic signatures between CI and HC groups. **a**, Volcano plot showing the upregulated (red) and downregulated (blue) metabolites in the CI group. **b**, VIP (Variable Importance in Projection) plot showing the top differential metabolites. **c**, KEGG enrichment analysis showing the primary metabolic pathways involved in the pathological process of CI. The color and size of the circles represent the significance and impact of the pathways, respectively. *P*_value and *P*_adjust for enriched paythways were listed

To further investigate the systemic metabolic changes associated with cerebral infarction, we performed untargeted metabolomic profiling on serum samples. A total of 16,144 peaks were detected, resulting in the identification of 2,550 unique metabolites (Table 3). Our comparative analysis revealed a distinct metabolic signature in CI patients; specifically, we identified 445 differentially expressed metabolites, of which 159 were significantly upregulated and 286 were downregulated compared with the HC group (Fig. 3a).

**Table 3:** Summary of metabolites identified in the metabolomic analysis

Ion mode	All peaks	Identified metabolites	Unidentified	Metabolites in Library	Metabolites in KEGG	P_value	P_adjust
pos	8885	1222	7663	964	439	< 0.05	< 0.05
neg	7259	1328	5931	1138	493	< 0.05	< 0.05
mix	16,144	2550	13,594	2102	932	< 0.05	< 0.05

Among the top differential metabolites, we observed a significant increase in the levels of Radicinin, NNAL-N-glucuronide, and clopidogrel carboxylic acid in CI patients. Conversely, D-3-phenyllactic acid sulfate, vanillin-4-sulfate and 2-hydroxyacetophenone sulfate were markedly decreased (Fig. 3b). To understand the functional implications of these shifts, we performed Kyoto Encyclopedia of Genes and Genomes (KEGG) pathway enrichment analysis. The results demonstrated that these altered metabolites were primarily involved in glycerophospholipid metabolism, phenylalanine metabolism, and caffeine metabolism (Fig. 3c). Notably, the enrichment in choline metabolism, suggests a broad metabolic reprogramming characterized by lipid and amino acid dysregulation in the acute phase of CI was also observed.

### Proteomic analysis identifies unique protein signatures associated with inflammatory and immune activation in CI

**Fig. 4:**
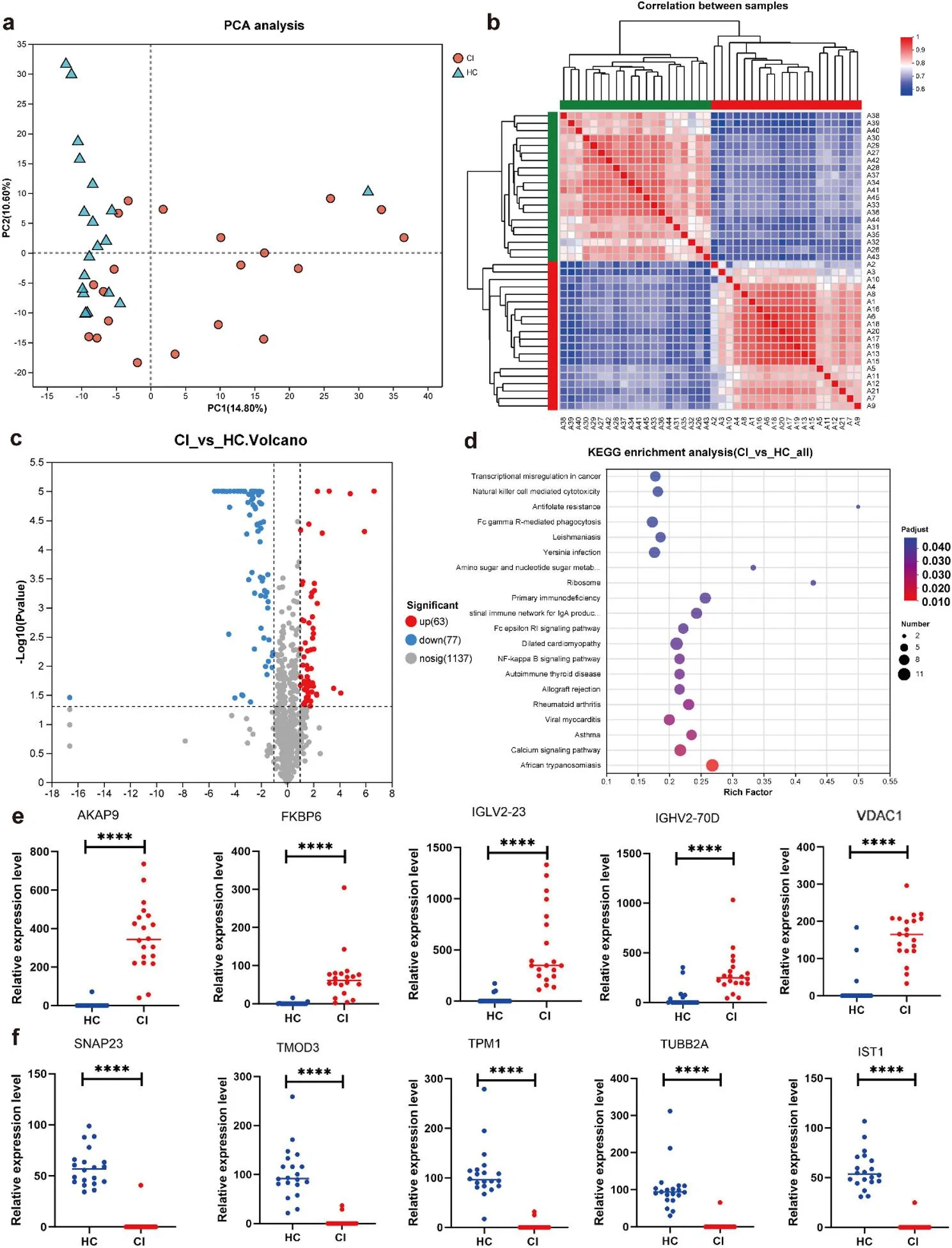
Proteomic characterization and differential expression analysis. **a**, Principal Component Analysis (PCA) score plot illustrating the overall proteomic separation between CI and HC groups. **b**, Heatmap of sample-to-sample correlation analysis demonstrating the consistency within groups. The heatmap was generated using the pheatmap R package. Sample clustering (column dendrogram) was constructed first using the complete linkage method based on Euclidean distance, and hierarchical clustering was then applied to the normalized protein expression values across all identified proteins **c**, Volcano plot showing the overall distribution of differentially expressed proteins (DEPs). **d**, Kyoto Encyclopedia of Genes and Genomes (KEGG) pathway enrichment analysis identifying significantly activated biological processes. **e**, Top five most significantly upregulated proteins. **f**, Top five most significantly downregulated proteins. ****P< 0.0001

Next, we characterized the serum proteomic landscape to identify key protein drivers of the disease state. A total of 1,299 proteins were successfully identified across all samples. Principal Component Analysis (PCA) demonstrated a clear separation between the CI and HC groups, indicating that CI patients possess a unique proteomic fingerprint (Fig. 4a, b). Notably, one HC sample appeared as a moderate outlier in the PCA plot, which may reflect an underlying subclinical inflammatory state. We identified 140 differentially expressed proteins (DEPs), consisting of 63 upregulated and 77 downregulated proteins in the CI cohort (Fig. 4c).

To elucidate the biological pathways influenced by these proteomic changes, we conducted KEGG enrichment analysis. We found that the identified DEPs were significantly associated with the activation of the nuclear factor kappa B (NF-κB) signaling pathway, calcium signaling, and multiple pathways intrinsically linked to inflammation and the immune response (Fig. 4d). Detailed analysis of the DEPs revealed that AKAP1, FKBP6, and VDAC1 were among the most significantly upregulated proteins, whereas SNAP23, TMOD3, and TPM1 were the most significantly downregulated (Fig. 4e, f). These findings suggest that the serum proteome of CI patients reflects a state of systemic inflammation and cellular stress, potentially contributing to the secondary injury cascade following the initial ischemic event. 

### Gut dysbiosis correlates with altered serum lipid profiles and the accumulation of neurotoxic metabolites

**Fig. 5:**
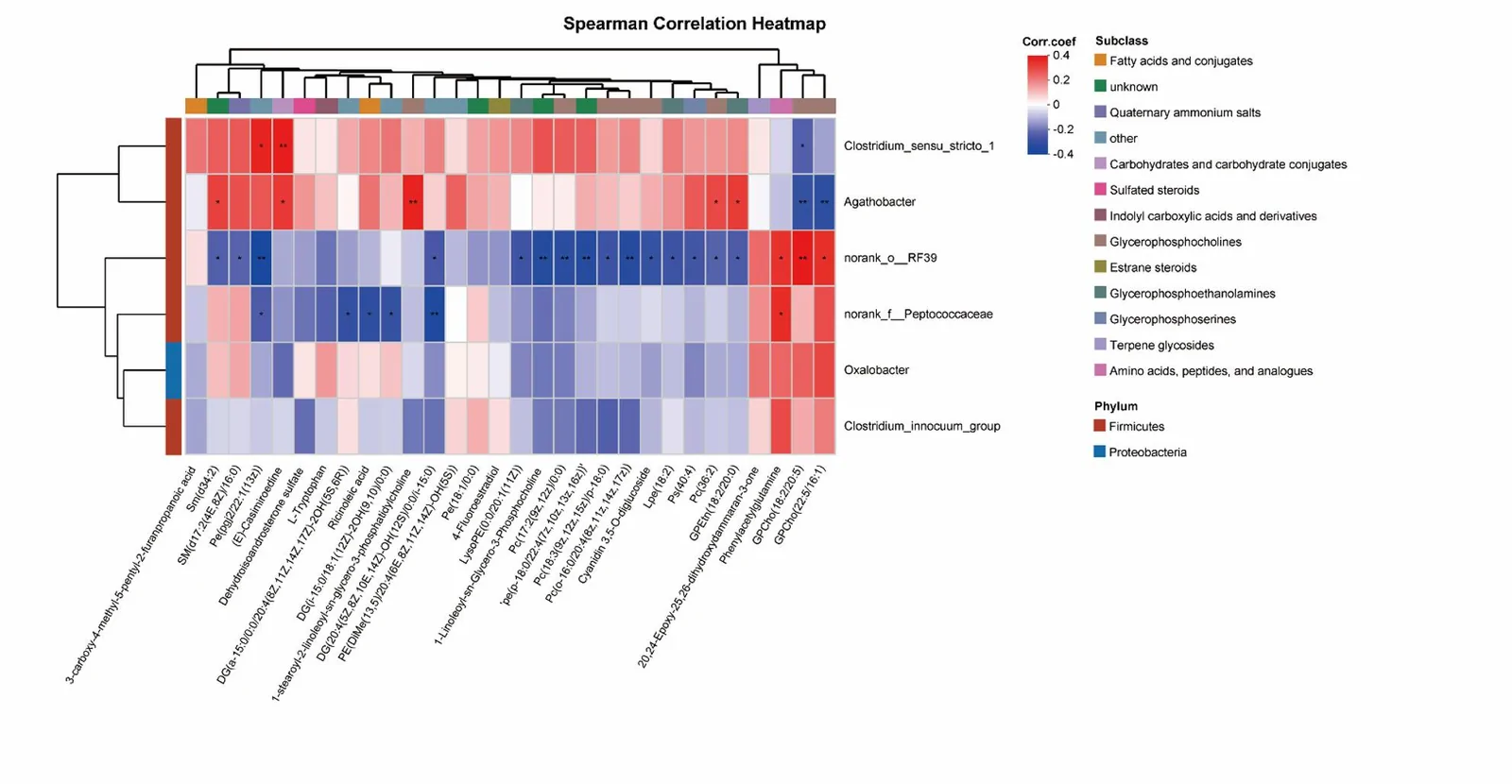
Correlation analysis between differential gut microbiota and serum metabolites. The row (metabolites) and column (genera) dendrograms were constructed using complete linkage clustering based on Euclidean distance. LPE, lysophosphatidylethanolamine; PC, phosphatidylcholine. **P< 0.01; *P < 0.05

To elucidate the potential functional impact of gut dysbiosis on the systemic metabolic environment, we performed a correlation analysis between the differential microbial genera and serum metabolites. We observed a robust association between the CI-enriched genus g_norank_o_RF39 and several lipid species; specifically, g_norank_o_RF39 was positively correlated with glycerophosphocholine (GPCho) (22:5/16:1), GPCho (18:2/20:5), and the uremic toxin phenylacetylglutamine (*P* < 0.05) (Fig. 5). Conversely, this genus showed a significant negative correlation with LPE (18:2) and PC (36:2). Interestingly, the health-associated genus Agathobacter exhibited a nearly opposite correlation pattern, being positively associated with the protective lipid species depleted in CI patients. These findings suggest that the gut microbiota may influence the host metabolic state by modulating glycerophospholipid profiles and the production of neurotoxic metabolites.

### Differential serum metabolites are strongly associated with proteins involved in vascular and inflammatory pathways

**Fig. 6:**
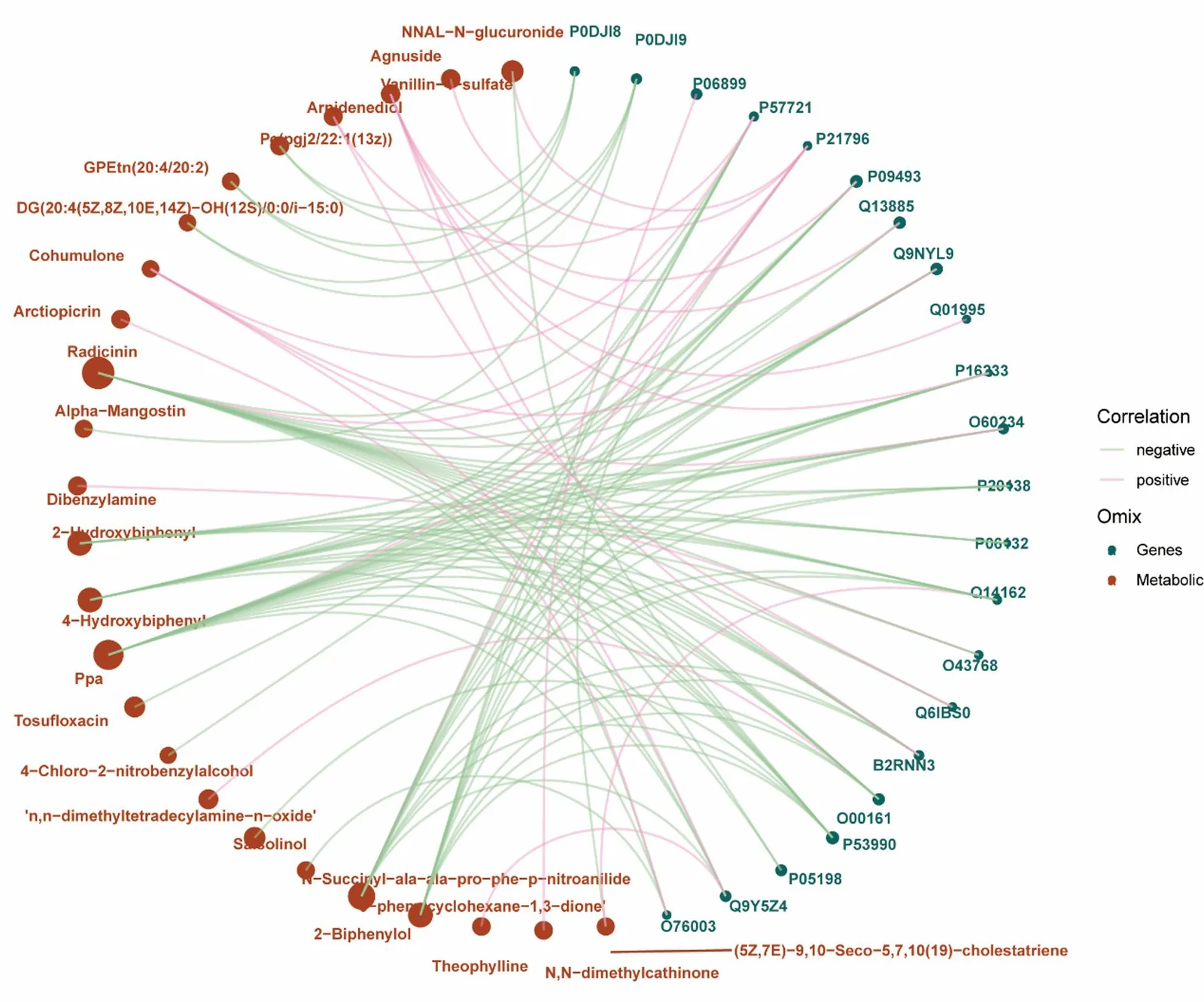
Correlation analysis between differential serum metabolites and proteins in CI patients

To further bridge the gap between metabolic shifts and functional protein expression, we conducted a metabolome–proteome integration analysis. We identified strong correlations between key differential metabolites and proteins involved in the pathological progression of stroke. Specifically, upregulated metabolites in CI patients, including Radicinin, NNAL-N-glucuronide, and phenylpropionic acid (PPA), were found to be strongly associated with a cluster of differential proteins (including Q76003, Q60234, P21796, and P53990) (all Spearman |r| > 0.6, FDR q < 0.05) (Fig. 6). Functional annotation indicated that these correlated protein-metabolite pairs are primarily involved in CI-related inflammatory responses and vascular endothelial dysfunction pathways. This integrated landscape suggests a synergistic relationship where metabolic disturbances may drive, or be driven by, the activation of systemic inflammatory and vascular signaling cascades in cerebral infarction.

## Discussion

In this study, we employed an integrated multi-omics approach to characterize the complex interplay between the gut microbiota, serum metabolome, and serum proteome in patients with cerebral infarction (CI). Our findings demonstrate that CI is associated with a distinct molecular fingerprint characterized by microbial dysbiosis, significant reprogramming of lipid and amino acid metabolism, and the activation of systemic inflammatory proteomic pathways. By bridging these layers, our integration analysis revealed interconnected microbiota–metabolite–protein axes that may contribute to CI pathogenesis via the gut–brain axis.

While the global structure of the gut microbiota (alpha and beta diversity) remained relatively stable, we identified critical shifts in specific taxonomic clusters. The significantly lower GMHI and higher MDI in CI patients underscore a state of microbial imbalance. Alpha and beta diversity metrics capture overall community structure (richness, evenness, compositional similarity), whereas GMHI and MDI are targeted indices reflecting the relative balance of specific health- versus disease-associated taxa. This pattern is biologically analogous to many disease states (e.g., early metabolic disease, hypertension) where shifts in keystone taxa occur without dramatically altering global diversity. Notably, the enrichment of *g_norank_o_RF39* and *Oxalobacter* in CI patients, contrasted with the depletion of *Agathobacter*, suggests a shift away from a homeostatic microbiome. *Agathobacter* is a well-known producer of butyrate, a short-chain fatty acid (SCFA) crucial for maintaining intestinal barrier integrity and suppressing systemic inflammation [15–18]. Furthermore, the negative correlation between *Akkermansia* and NIHSS scores is particularly noteworthy. As a “sentinel” of the gut barrier, *Akkermansia* may play a neuroprotective role by dampening the systemic inflammatory response, consistent with its association with milder neurological deficits in our cohort [19–21].

Our metabolomic analysis highlighted significant alterations in glycerophospholipid and phenylalanine metabolism. The positive correlation between *g_norank_o_RF39* and the uremic toxin phenylacetylglutamine (PAGln) is a key finding. Recent literature has identified PAGln as a gut-microbiota-derived metabolite that enhances platelet reactivity and thrombosis potential through adrenergic receptor signaling [22–24]. The concurrent reduction in protective lipids, such as LPE (18:2) and PC (36:2), further suggests that CI-associated dysbiosis promotes a pro-thrombotic and pro-inflammatory metabolic milieu. These metabolic shifts likely serve as a critical bridge through which gut dysbiosis influences cerebrovascular outcomes. However, we must note that clinical interventions during acute stroke management introduce unavoidable confounding effects. For instance, clopidogrel carboxylic acid was identified as a top upregulated metabolite in the CI group, indicating that antiplatelet therapy had been initiated. These treatment-induced metabolic and proteomic alterations may overlay the baseline pathology of cerebral infarction. Future studies controlling for immediate post-onset medication or collecting samples strictly hyper-acute (pre-treatment) are necessary to uncouple disease pathophysiology from therapeutic confounding.

The serum proteome of CI patients revealed a state of heightened cellular stress and inflammatory activation, evidenced by the upregulation of VDAC1 (associated with mitochondrial apoptosis) and AKAP1. The activation of NF-κB and calcium signaling pathways further reflects the systemic inflammatory cascade triggered by ischemic insult [25–28]. Our integration analysis identified strong associations between upregulated metabolites (e.g., Radicinin, PPA) and proteins involved in vascular endothelial dysfunction. This suggests that the post-stroke serum environment is saturated with small molecules that may exacerbate vascular permeability and neuroinflammation, potentially through the downregulation of structural or trafficking proteins like SNAP23 and TPM1.

The strength of this study lies in the simultaneous integration of three molecular layers. Although, the relatively small sample size of our cohort (20 CI patients and 20 healthy controls) limits the statistical power to discover subtle multi-omics interactions and increases the risk of false-positive associations. Therefore, the identified microbiota-metabolite-protein regulatory axes should be interpreted as hypothesis-generating, and further large-scale longitudinal cohorts are strictly warranted to validate these findings. The multi-omics signatures identified in this study have potential translational value across several dimensions. First, as diagnostic biomarkers, the combination of GMHI/MDI with circulating metabolites (PAGln, LPE (18:2), PC (36:2)) could form a multi-omics panel for non-invasive CI screening. Second, as prognostic indicators, the association between *Akkermansia* abundance and lower NIHSS scores suggests that microbiota composition may correlate with neurological severity. Third, as therapeutic targets, the *g_norank_o_RF39* → PAGln → inflammatory signaling axis identified here suggests testable intervention strategies, including probiotic restoration of butyrate-producing taxa and pharmacological modulation of the PAGln–β2-adrenergic receptor axis.We propose a model where CI-associated dysbiosis (e.g., loss of *Agathobacter*, gain of *g_norank_o_RF39*) alters the availability of circulating metabolites like PAGln and glycerophospholipids.

These metabolic changes are intimately linked to a proteomic shift toward an inflammatory and pro-thrombotic state (NF-κB activation, VDAC1 upregulation). This integrated “Microbiota-Metabolite-Protein” axis provides a holistic view of the systemic environment in CI, offering a broader range of potential biomarkers and therapeutic targets than single-omics studies.

## Conclusion

In summary, our study delineates a clear “gut–brain–metabolism” axis in cerebral infarction. The identified microbial markers, particularly the ratio of dysbiosis and the abundance of *Akkermansia*, correlate significantly with clinical severity. Multi-omics integration reveals that specific gut microbiota are closely associated with key serum metabolites and proteins involved in inflammation, vascular function, and neurological impairment. These microbiota–metabolite–protein networks may serve as novel diagnostic biomarkers and therapeutic targets for cerebral infarction.

## Data Availability

All data generated or analyzed during this study are included in this published article. All raw data for microbial sequencing, proteomics, and metabolomics have been deposited in NCBI (https://www.ncbi.nlm.nih.gov/bioproject/PRJNA1456750/), ProteomeXchange (https://proteomecentral.proteomexchange.org/ui? pxid=PXD077506), and MetaboLights (https://www.ebi.ac.uk/metabolights/MTBLS14356), respectively, and are publicly accessible.

## Abbreviations

CI: Cerebral ischemic stroke
HC: Healthy controls
BMI: Body mass index
GMHI: Gut microbiome health index
MDI: Microbiome dysbiosis index
PCR: Polymerase chain reaction
VIP: Variable importance in projection
NIHSS: National Institutes of Health Stroke Scale
SCFA: Short-chain fatty acid
PAGln: Phenylacetylglutamine
VDAC1: Voltage-dependent anion channel 1
AKAP1: A-kinase anchor protein 1
SNAP23: Synaptosome-associated protein 23
TPM1: Tropomyosin 1
TMOD3: Tropomodulin 3
